# Feasibility of introducing integrated disease surveillance and response into curricula of public health training institutions in Sierra Leone: the process and lessons learnt

**DOI:** 10.3389/fpubh.2024.1467402

**Published:** 2024-12-18

**Authors:** Charles Njuguna, Abdul Mbawa, Ian Njeru, Innocent Bright Nuwagira, Mohamed Vandi, Joseph Sam Kanu, James Sylvester Squire, Aminata Tigiedankay Koroma, Ade Renner, Robert Musoke, Wilson Gachari, Victor Caulker, Jane Githuku, Gerald Shambira, Boukare Bonkoungou, Ambrose Talisuna, Etien Luc Koua, Dick Chamla, Zabulon Yoti, Abdou Salam Gueye

**Affiliations:** ^1^World Health Organization Regional Office for Africa, Brazzaville, Republic of Congo; ^2^College of Medicine and Allied Health Sciences, Freetown, Sierra Leone; ^3^World Health Organization Country Office, Freetown, Sierra Leone; ^4^Ministry of Health, Freetown, Sierra Leone; ^5^Independent Consultant, Freetown, Sierra Leone; ^6^World Health Organization Liaison Office to African Union, Addis Ababa, Ethiopia

**Keywords:** integrated, disease, surveillance, response, pre-service, curriculum, Sierra Leone

## Abstract

**Introduction:**

Response to public health emergencies is a big challenge in African countries due to inadequate workforce. Integrated Disease Surveillance and Response (IDSR) is a strategy implemented by African member states of WHO to strengthen capacity for disease surveillance and response at all levels. Despite successful implementation of IDSR in most countries, one of the challenges that persists is that of inadequate trained workforce competent enough for public health surveillance. Introducing IDSR pre-service curriculum in public health training institutions has therefore been recommended by WHO as one of the strategies to sustainably address the human resource challenge. We report on the process and lessons learnt in Sierra Leone which was the first country to implement the recommendation.

**Methods:**

This was a descriptive study where the process of introducing and implementing IDSR pre-service curriculum in Sierra Leone was documented from 2018 to 2024. Data was collected through observation, documentation and analysis of key processes that took place. These processes included, *inter alia*, advocacy with public health training institutions, development of the IDSR pre-service curriculum and incorporation of the curriculum into the existing training programs of colleges and universities.

**Results:**

IDSR preservice curriculum was developed and successfully introduced in eight targeted public health training institutions in Sierra Leone from September 2021. Training content was adapted from the 3rd Edition of IDSR technical guidelines developed by WHO in 2019. As at February 2024, more than 4,200 students had started taking IDSR modules in the eight institutions with 2,108 having completed and graduated. During the process, we learned that key enablers to success were government support, good advocacy with the training institutions and training of lecturers on IDSR. Main challenges were the long process of curriculum approval by training institutions and handling of big classes of students without adequate training materials.

**Conclusion:**

Introducing IDSR into the preservice curricula of public health training institutions is feasible and can provide a reliable and continuous supply of a trained workforce ready to be utilized for IDSR in Africa. Successful implementation requires advocacy with training institutions as well as regular monitoring of the implementation to maintain good quality.

## 1 Introduction

The International Health Regulations (IHR 2005) are an instrument of international law that is legally-binding on 196 countries, including the 194 WHO Member States (1). The IHR 2005 became operational in June 2007 and require State Parties to develop minimum core public health capacities to detect, assess, notify and respond to public health events in order to strengthen national and global health security (2, 3). The IHR also require State Parties to strengthen core surveillance and response capacities at the primary, intermediate and national level, as well as at designated international ports, airports and ground crossings (4, 5).

In order to support countries in Africa to build their public health surveillance and response capacities, the World Health Organization developed the Integrated Disease Surveillance and Response (IDSR) strategy (6). The IDSR strategy, which acts as one of the vehicles for implementing IHR, was first adopted in 1998 although the first technical guidelines on IDSR implementation were released in 2001. The IDSR technical guidelines were later revised in 2010 (second edition) in order to allow compliance to the revised IHR (2005) guidelines, specifically to increase the scope of public health events under surveillance, increase core public health capacities in member states and establish formal IHR coordination structures (7). The guidelines were again revised in 2019 (third edition) in compliance with the revised regional IDSR strategy (2020–2030) and in order to incorporate new diseases/conditions/events, technologies, approaches and lessons learnt in responding to past outbreaks (8, 9).

Among the consistent challenges hindering IDSR implementation in member states is lack of adequate trained workforce, sometimes due to high staff turnover (10–13). For example, assessment of key IDSR performance indicators among African member states has in the past revealed low coverage of IDSR training among staff (14). Due to financial challenges, most African states including Sierra Leone train health workers on IDSR using a phased in-service training approach which is costly and takes time to train the required numbers (15). In the initial training, most countries focus on having at least one health worker (e.g., IDSR focal person) trained per health facility with the hope that the trained personnel will train others which most of the times does not happen. Other critical staff especially clinicians and laboratory personnel who play a critical role in IDSR are often trained late or not at all. At least 95% of IDSR reporting health facilities in Sierra Leone have one or more technical staff trained on IDSR third edition guidelines and tools (16).

To address, the IDSR training challenge, WHO African Regional Office (WHO AFRO), proposed a change in tact by recommending several solutions such as eLearning and introduction of IDSR into preservice curriculum of public health training institutions (14). The latter involves integrating IDSR training into pre-service curricula of medical training institutions, an approach that was expected to be effective and sustainable (17). In deed inadequacy of pre-service training curricula to prepare medical and public health students for their jobs is well documented with calls for continuous review of curricular in response to changing needs in the health sector (18–21). While eLearning for IDSR has been created by WHO and is now available to health workers in most African countries (22), preservice training is yet to take off.

Sierra Leone is among the countries with the highest shortage of health care workers and had the lowest density of medical doctors in West Africa in 2020 at only three physicians per 100,000 individuals (23, 24). Specialized health workers are therefore mainly found in regional and specialized hospitals in urban areas. Health services in peripheral health units are provided by less specialized health professionals such as state registered nurses, maternal child health aides, environmental health workers and midwives (25). These professionals also play a critical role in surveillance and IDSR mostly in identification, reporting and notification of priority health diseases, conditions and events.

Curriculum development and update in training institutions is a continuous process that is necessitated by many factors including technological advancement, demands by students, society expectations, the need for professionalism in business, academic research to revamp the economy, and government expectations, among others (26). There is therefore an opportunity to introduce IDSR to the curriculum of public health training institutions. Based on the health worker shortage and willingness to participate in the study, Sierra Leone was selected by WHO to pilot the integration of IDSR training into the curricula of higher learning institutions offering medical related training.

This paper documents the process used and the lessons learnt during the integration of IDSR into the curriculum of public health training institutions in Sierra Leone. Being the first country to do so, the insights obtained will be very helpful to other African member states who may desire to incorporate IDSR or other specialized training in medical training institutions.

## 2 Materials and methods

### 2.1 Design and setting

This was a descriptive study where the process of integrating IDSR pre-service curriculum into eight public health training institutions in Sierra Leone was observed and documented from 2018 to 2024. Introducing the IDSR curriculum into public health institutions was done as one of the strategies recommended to the Ministry of Health to increase the country’s capacity on IDSR and preparedness and response to public health emergencies. The curriculum was developed using the 3^rd^ Edition of IDSR guidelines that were released by WHO AFRO in 2019 and adapted by Sierra Leone in the same year. WHO Sierra Leone supported the Ministry of Health in this process by providing technical support.

### 2.2 Data collection and analysis

Data was collected through documentation of key processes that took place during the development and implementation of the IDSR preservice curriculum. The reports of these processes were then used to extract data and information for this paper. The reports were supplemented by information from key informants. The key processes included advocacy with public health training institutions, review and adaptation of the generic WHO IDSR training materials and development of the draft IDSR pre-service curriculum by two consultants. The draft curriculum was reviewed by the participating public health institutions who provided useful comments and recommendations to the consultancy team.

The final curriculum was reviewed and approved by the Ministry of Health and Sanitation, (MOHS), Ministry of Technical and Higher Education (MTHE) and the various public health training institutions. The curriculum was then integrated into the existing curricula of the public health training institutions and this was then followed by training of trainers (lecturers) and commencement of the training for the students.

During the implementation process, qualitative data was mainly captured as activity reports using standardized activity reporting templates that captured, *inter alia*, objectives, justification, methodology, participants and outputs/outcomes. Quantitative data was collected during field visits to the institutions to monitor implementation. This data was collected electronically using Open Data Kit (ODK) and analyzed using Microsoft Excel. The data collected included, *inter alia*, the number of students enrolled by department, course and modules, the number that had completed each module as well as the challenges and lessons learnt.

The key overall outcomes of the analysis were the various processes and milestones, content of the curriculum and time taken for development and implementation of the curriculum.

## 3 Results

The study focused on finding out how the process of IDSR curriculum development and implementation was carried out as well as the number of beneficiaries. Below were the findings.

### 3.1 The implementation process

The process to introduce IDSR into the preservice curriculum of public health institutions started with advocacy and consultative meetings between WHO Country Office, Sierra Leone Ministry of Health and eight leading medical training institutions. These institutions were: University of Sierra Leone College of Medicine and Allied Health Sciences (COMAHS), Njala University, University of Makeni, Ernest Bai Koroma University of Science and Technology, School of Midwifery Bo, School of Midwifery Makeni, Eastern Technical University of Sierra Leone and National School of midwifery, Freetown. Once buy-in was obtained, two senior lecturers (led by the head of community health department) from COMAHS, University of Sierra Leone (the main health training institution) were contracted in February 2018 to conduct a desktop review of existing MOH and WHO IDSR guidelines and training materials and propose which content should be incorporated into the IDSR preservice curriculum of public health training institutions. They were also tasked to develop the draft curricula for preservice as well as for in-service health workers.

Once the draft curricula were developed by the consultants, a Technical Working Group (TWG) comprising of subject matter experts was formed in July 2019 to review and validate the curricula. The TWG comprised of participants from MOHS, WHO and representatives from the eight training institutions. The validation of the curricula by TWG was done in July 2019 and this was followed by training of lecturers between February and August 2020. Since the content of the training materials to be used in the institutions was largely the same as the one for the MOH, the lecturers were trained together with the national IDSR Trainers of Trainers (TOTs) who were also being trained for the roll out of the third edition of IDSR.

The official launch of the curricula was done in October 2020 (27) and it brought together stakeholders from Ministry of Health, Ministry of Technical and Higher Education and technical and administrative leads from the targeted public health training institutions and universities. The purpose of the launch forum was to appraise stakeholders about the content of the curriculum and seek support for its implementation. The forum was also an opportunity for WHO to formally hand over the curriculum to MOHS, MTHE and the training institutions.

**Figure 1:**
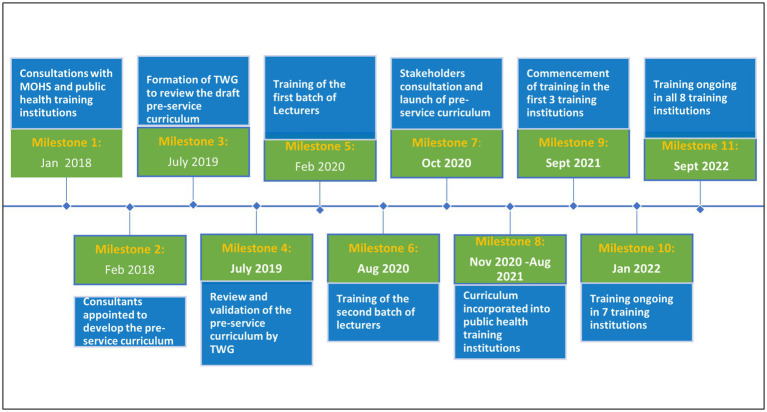
Milestones in the development and implementation of IDSR pre-service curriculum in Sierra Leone.

Following the launch of the IDSR curriculum, the training institutions engaged their senates and governing boards for approval and integration of the IDSR curriculum into their relevant preservice curricula. This process occurred between November 2020 and August 2021. By September 2021, Eastern Technical University, UNIMAK, EBKUST had commenced training while Midwifery school Bo, Midwifery school Makeni, University of Sierra Leone, and National School of Midwifery Freetown had commenced training by January 2022. Figure 1 summarizes the various milestones in the curriculum development and implementation process.

### 3.2 The curriculum

**Figure 2:**
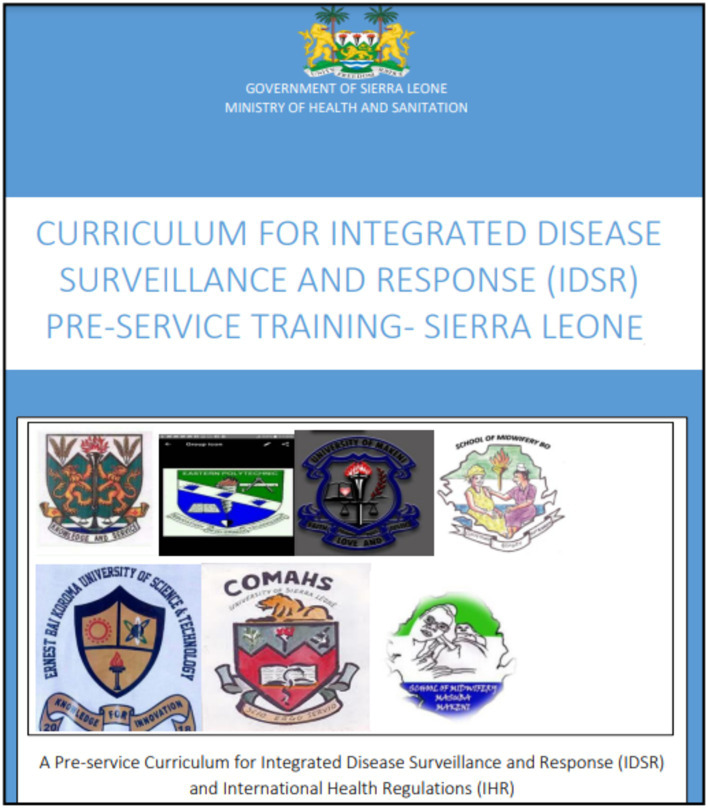
Pre-service curriculum for IDSR in Sierra Leone.

The consultants reviewed the 3rd Edition of IDSR Technical guidelines and training modules that were developed by WHO AFRO in 2018 (9). Upon consultations with WHO and MOHS, the generic IDSR training modules that were developed for use by Ministries of Health in the African region were found to be adequate in terms of content and were therefore adapted in their current format into developing the curriculum for the public health training institutions. Two approaches of incorporating IDSR training modules were proposed and implemented: a pre-service curricular for public health students (Figure 2) and an in-service four- month certificate and diploma curriculum for employed public health workers (28).

The target audience for the pre-service training were trainees and students enrolled in health training undergraduate and post graduate programs in the various institutions. These included community health, general surgery and medicine (MBCHB), nursing (diploma and bachelor), midwifery, laboratory sciences, public health, environmental health, health promotion, rural development studies and other allied health sciences. The IDSR-IHR curriculum was added as a distinct module and added into the existing diploma, undergraduate, and postgraduate programs/courses at the discretion of the institutions. The training duration depended on the study period of the various courses in the training institutions. At undergraduate level, the IDSR-IHR module was added into the existing courses and the 11 sub modules spread over the study period. However, whether the modules were covered over a semester, or several semesters or years was left at the discretion of the training institution. For the postgraduate level studies, the recommendations was for the IDSR module to be added to the courses and the IDSR sub modules spread out over the life span of the postgraduate diploma and master’s degree programs. Just like the under graduate courses, the training institutions were given the leeway to decide whether the IDSR sub modules would be covered over a semester or several semesters.

The training duration at each level of cadre was left open to the institutions as long as the minimum 43 h of credit were met (Table 1). Where there had been successful integration of core modules at undergraduate level training, institutions were given the option to offer only the advanced modules at postgraduate level. Certain exemptions could be made for students that had undergone a certified course in IDSR-IHR.

**Table 1:** Pre-service IDSR-IHR modules for undergraduate and graduate level.

Course code	Course description	Credit hours
IDSR Intro	Introduction to IDSR-IHR	5 h
IDSR-1	Module 1: Identify and record cases of priority diseases, conditions and events	5 h
IDSR-2	Module 2: Report Priority Diseases, Conditions and Events	4 h
IDSR-3	Module 3: Analyze and Interpret Data	3 h
IDSR-4	Module 4: Investigate, Confirm Suspected Outbreaks and other public health events	4 h
IDSR-5	Module 5: Prepare to Respond to Outbreaks and Other Public Health Events	4 h
IDSR-6	Module 6: Respond to outbreaks and other public health events	3 h
IDSR-7	Module 7: Risk Communication	3 h
IDSR-8	Module 8: Monitor, Supervise, Evaluate, and provide feedback to Improve Surveillance and Response	3 h
IDSR-9	Module 9: Electronic Integrated Disease Surveillance and Response (e-IDSR)	3 h
IDSR-10	Module 10: Tailoring IDSR to Emergency or Fragile Health System Contexts	3 h
IDSR-11	Module 11: IHR at Points of Entry (POE)	3 h
UGFP	Under Graduate Field Practicum (UGFP)	2 Weeks
PGFP	Post Graduate Field Practicum (PGFP) and report as part of final year dissertation	2 weeks

In addition to the didactic sessions, a field practicum was recommended to the institutions. For the undergraduate studies, the recommendation was for the trainees to be attached to District Medical Officer or District Surveillance Officer who would take them through some orientation of the district health management system as well as introduce them to surveillance activities in the field sites such as periphery health facilities level. The trainees can work as individuals or in groups and are expected to complete at least two core activities for learning (e.g., describe a surveillance system, analyze a data set, attend surveillance meetings) and to prepare a report before the end of the attachment period. For the postgraduate programs, the recommendation was for the students to carry out field work as part of the research work in order to have some experience in applying the practical aspect of the modules learnt.

### 3.3 Number of students trained

Tracking on implementation was done using an electronic ODK tool that was completed through monitoring visits to the training institutions. By March 2024, more than 4,200 students from all institutions had started undertaking the IDSR courses with at least 2,108 (50%) having completed and graduated. Of those who had graduated, about 29% had managed to secure employment. Table 2 shows the number of students that had started or completed the IDSR training by training institution, department, and program.

**Table 2:** Number of students trained on IDSR/IHR by institution as of March 2024.

Name of public health training institution/ university		Departments/Faculties	IDSR/IHR fully integrated into curriculum	Number of trained lecturers	Year training started	Program	Number of students who have graduated
1	Ernest Bai Koroma University (EBKURST)	Public Health; Nursing	Yes	15	2021	BSC & Diploma	856
2	University of Makeni	Public Health; Development studies; Mass communication	Yes	8	2021	BSC & Diploma	550
3	Njala University	Public Health	Yes	9	2022	Not available	Not available
4	Eastern Technical University	Public health; Medical lab; School of Nursing	Yes	11	2021	BSC; Higher National Diploma; Diploma	169
5	National School of Midwifery, Freetown	Midwifery	Yes	5	2021	Diploma	150
6	School of midwifery, Makeni	Midwifery	Yes	6	2022	Diploma	155
7	School of Midwifery, Bo	Midwifery	Yes	6	2021	Diploma	143
8	College of Medicine and Allied Health Sciences (COMAHS)	Nursing; Community Health	Yes	6	2021	MPH, MBCHB, BSc	85
	Total						2,108

## 4 Discussion

Due to climate change and other factors, there is increased frequency of emerging and re-merging diseases (such as Ebola, Marburg, Dengue, Mpox, Cholera) globally that require rapid detection and response (29). This calls for competent personnel including doctors, nurses, laboratory scientists, epidemiologists, public health officers and environmental health officers. Integrating IDSR into the curriculum of public health training institutions is one strategy that is expected to ensure that personnel who qualify from these institutions have the relevant skills and competences to detect and respond to the emerging and re-emerging diseases on time (30).

Despite the benefits expected from it, the concept of having IDSR modules in public health training institutions had not yet taken off in Africa until Sierra Leone demonstrated that this is feasible. The actual implementation started in 2021 (27) and by March 2024, the country had succeeded in integrating IDSR into the preservice curriculum of 8 public health institutions where at least 2,100 students had graduated after taking all the modules. The process took longer than initially planned because of the COVID-19 pandemic disruption but several lessons were learnt on successful implementation including the enablers and challenges during the process.

### 4.1 Enablers of success

Several factors contributed to the successful rollout of the IDSR curriculum in Sierra Leone. Buy-in, ownership and leadership by the government ministries and public health training institutions is critical and this was successfully done in Sierra Leone. This was achieved through high level of engagement with the Ministry of Health, the Ministry of Technical and Higher Education and the eight public health training institutions to ensure there was implicit understanding of the importance of IDSR and IHR skills in the graduates trained in medical institutions.

During the development of the curriculum, the country also engaged two consultants from one of the targeted public health training institutions that have a successful public health program. The consultants guided the curriculum development process and provided support in the initial phase of implementation. In addition, well trained and competent lecturers are key to the roll out of IDSR curriculum. Lecturers from the eight training institutions were therefore involved during the launch, training and roll out of the 3^rd^ Edition of IDSR guidelines in 2019 and 2020 (31). This training was integrated with the national IDSR TOTs training, and this provided an opportunity for the lecturers to interact with seasoned IDSR practitioners who have vast experience in IDSR implementation.

During the training of the lecturers, one key factor that was considered when introducing the curriculum was how to deliver the training to a big class size in the public health training institutions. Traditionally, IDSR content is delivered through 5-days in-service training sessions, using brief PowerPoint presentations, individual exercises and small group discussions. Participants also use printed participant manuals, flip charts, graph paper, computers and printed slides. When training large groups of students, it was agreed to modify the training approach to focus more on PowerPoint presentations and class discussion due to scarcity of resources for printed materials.

Whereas, the IDSR in-service training takes about 5 days because of time constraints and the fact that the health workers are conversant with most of the content, the pre-service curriculum was spread out over 1 to 4 years depending on the courses in the public health training institutions. This approach worked well as it provided adequate time for learning and practicing of the many exercises and concepts contained in the 11 modules and the field practicum. However, it is important to note that this can be a counterproductive for some students who may find it more difficult to relate the content of the various modules that are taught several months or years apart.

Another important point to keep in mind during implementation of preservice IDSR training is that it may not completely replace the need for in-service training as those already employed will still need refresher trainings to get new updates on major changes related to IDSR that may be carried in future editions and also to cater for attrition of knowledge. However, preservice training helps to equip the trainees with basic competencies and understanding to perform core IDSR functions of detection, reporting, investigation, and response.

Finally, successful implementation of IDSR preservice curriculum depends on availability and retention of trainers. Public health training institutions should therefore ensure that more lecturers are trained on a continuous basis. The Ministries of Health should also ensure that lecturers of the participating institutions are regularly updated on any changes in the curriculum including through refresher workshops as necessary. There is also need to maintain some members of the consulting group and the TWG to provide regular updates to the various institutions on any modifications on the IDSR/IHR guidelines from the MOH and WHO. Having regular review meetings with the training institutions would also help in resolving some of the challenges.

While there was no literature available online for experience in introducing IDSR curriculum, the above enablers of successful curriculum implementation are in line with what other researchers have found for other courses in general. A critical understanding of a curriculum change is very important to ensure all stakeholders (leaders, teachers, learners, interest groups etc.) understand its purpose and hence support its implementation (32, 33). Other important factors include availability of resource materials, technologies and facilities as well as right environment, culture, supervision, and regular assessments (34, 35).

### 4.2 Challenges during implementation

The process of curriculum development and commencement of training took almost 2 years because of several factors. First, the country stakeholders agreed to use the 3rd edition of IDSR guidelines and modules, and this were only launched by WHO AFRO in 2019 which was more than a year after the consultants were hired to develop the curriculum. Second, the process was affected by the COVID-19 pandemic which was declared as a Public Health Emergency of International Concern (PHEIC) on 30th January 2020 (36) and a pandemic on 11th March 2020 (37). The pandemic affected the implementation schedule as meetings and trainings had to be postponed and resources repurposed for outbreak response. Ideally with good planning, the whole process of curriculum development, validation and integration into university curricula should take about 1 to 2 years.

Third, the process was affected by the different levels of buy-in required from institutions of higher learning and the rigorous curriculum review and approval processes. While the IDSR curriculum was officially launched in October 2020, most institutions did not start training until September 2021 mostly because of long period of time taken to get approval from the senates and boards of the institutions. This administrative process could be shortened if advocacy and lobbying can start immediately the development of the curriculum begins.

Fourth, the delivery of the IDSR modules required equipment and materials such as computers, projectors, printers, photocopiers, white boards, and flip charts which were not adequately available in some of the training institutions. This therefore made projection of the slides and printing of materials such as the training exercises a big challenge. The training institutions were therefore advised to be innovative in addressing the challenges including use of bigger group class discussion where printing of materials was not possible due to high number of students.

Lastly, the number of trained lecturers that could offer training were relatively few in the beginning and some later left the institutions. This meant that training had to be organized again for the new lecturers. This challenge of staffing, together with inadequate resources also affected the planning and implementation of field practicum with Ministry of Health and is yet to take off in most institutions.

While some of the challenges were quite unique such as COVID-19 disruption, majority of the challenges have been experienced in other countries when implementing curriculum in universities. For example a study in Nigeria found some of the most common barriers for curriculum implementation to be underfunding, population explosion, quantity and quality of teaching staff, quality of students and time constraint (38). Other challenges encountered in other countries include lack of good understanding of the curriculum by staff and lack of collaboration by institutions and staff to share resources and knowledge (39, 40).

### 4.3 Limitations

Curriculum development, implementation and impact assessment takes time. Our study assessed part of this process and the time was not adequate to assess the impact of the trainees once they had graduated. For example, most of the graduates who had completed the IDSR studies were yet to get employment and had therefore not started service delivery. The study team will continue to follow up with the institutions and the graduates to be able to assess the impact further.

## 5 Conclusion

IDSR curriculum was successfully incorporated into eight public health training institutions in Sierra Leone. With continued engagement of these and other training institutions, a considerable proportion of the graduates in the medical field will have the basic understanding of IDSR upon graduation. If these graduates are absorbed into the labour market, they will be very instrumental in addressing the health worker shortage with regard to timely detection and response to public health emergencies which is the main goal of IDSR and IHR. Continued follow up and more research is required to assess the impact of the graduates on health delivery.

To successfully integrate IDSR into the public health training institutions, we recommend that countries conduct good advocacy with the training institutions and government to get buy-in. Availability of well-trained lecturers and training materials and resources should also be availed. Regular support and monitoring of the implementation by subject matter experts such as from MOH should also be conducted in the initial days for quality assurance of the training.

## Data Availability

The original contributions presented in the study are included in the article/supplementary material, further inquiries can be directed to the corresponding author.
